# Establishment and Application of a Multiplex PCR Assay for the Rapid Detection of *Rhizoctonia solani* Anastomosis Group (AG)-3PT, the Pathogen Causing Potato Black Scurf and Stem Canker

**DOI:** 10.3390/pathogens11060627

**Published:** 2022-05-29

**Authors:** Linda Iradukunda, Yan-Ping Wang, Oswald Nkurikiyimfura, Tian Wang, Li-Na Yang, Jiasui Zhan

**Affiliations:** 1 Marine and Agricultural Biotechnology Laboratory, Fuzhou Institute of Oceanography, Minjiang University, Fuzhou 350108, China; lidukunda@yahoo.fr; 2 Sichuan Provincial Key Laboratory for Development and Utilization of Characteristic Horticultural Biological Resources, College of Chemistry and Life Sciences, Chengdu Normal University, Chengdu 611130, China; yanpingwang46@126.com; 3 Institute of Plant Virology, Fujian Agriculture and Forestry University, Fuzhou 350002, China; nk.oswaldo@gmail.com; 4 Sichuan Vocational College of Chemical Technology, Luzhou 646000, China; wangtian484@gmail.com; 5 Department of Forest Mycology and Plant Pathology, Swedish University of Agricultural Sciences, 75007 Uppsala, Sweden; jiasui.zhan@slu.se

**Keywords:** potato, *Rhizoctonia solani* AG-3, multiplex PCR, molecular detection

## Abstract

*Rhizoctonia solani* anastomosis group 3 (AG-3) is the main causative agent of the soil-borne disease known as potato black scurf, which poses a huge threat to potato production. Rapid and accurate identification of *R. solani* AG-3 isolates in soil and potato seed tubers prior to planting is essential for good production. In this study, a multiplex PCR assay was established for the detection of *R. solani* AG-3. Two pairs of target-specific primers were designed from sequences for endopolygalacturonase and pyridoxine biosynthesis genes downloaded from GenBank. The main factors influencing PCR amplification, such as annealing temperature and primer concentration, were optimized. Results show that the proposed multiplex PCR assay is highly sensitive and specific for the target genes in the pathogen even when the DNA concentration is reduced to 20 fg/μL. The resulting calibration plot shows a linear relationship between electrophoretic band peaks and genomic DNA concentration (R^2^ = 0.98). The primer specificity was confirmed by applying them to other *R. solani* AG groups and plant pathogen species on which no amplicons were produced. Using the primers, we successfully detected small amounts of *R. solani* AG-3 present in soil and potato tuber samples. Taken together, the detection assay developed in this study has high sensitivity, strong specificity, and accuracy and can be used to detect and identify soil and potato seed tubers infected with *Rhizoctonia solani* AG-3.

## 1. Introduction

*Rhizoctonia solani* is a soil-borne plant pathogenic basidiomycete fungus with a wide geographical distribution and host range, resulting in significant yield and economic losses of many crops [1,2,3]. It is a species complex consisting of at least 13 anastomosis groups (AGs) varying in morphology, physiological characteristics, somatic compatibility, life history traits, host range and pathogenicity [4,5,6,7]. Based on ITS sequences, AGs (e.g., AG-2) can be subdivided into sub-groups (e.g., AG-2-2), and sub-groups can further be divided into subsets (e.g., AG-2-2 IIIB). Most AG groups are vegetatively incompatible with each other except for AG-BI which is vegetatively compatible with several other groups (AG-2, AG-8). Each AG group has one or more hosts [8,9,10]. For example, AG-2 can cause disease in a variety of host plants while AG-8 is usually specific to cereal crops.

*Rhizoctonia solani* can cause stem canker and black scurf on potatoes [11,12]. Stem canker is the symptom visible on potato sprouts and young growing shoots. It delays plant development, decreases stem number, and interrupts nutrient uptake [13,14,15]. Black scurf refers to the formation of small to large, dark black dots, i.e., sclerotia or survival stage of the pathogen, on the tuber surface, therefore, reducing tuber quality and economic value [8,16]. The disease leads to significant quantitative and qualitative losses of potato crops on the market with a value of up to 30% [16,17]. The Black scurf or sclerotia enable the pathogen to survive in plant debris for a long period of time due to enhanced tolerance to environmental stresses, such as low soil temperature and high soil moisture or help the pathogen in long-distance dispersal. 

Advances in serological and molecular assays have significantly facilitated the detection of *R. solani* [18,19,20]. The traditional assay for the pathogen is based on a visual inspection of disease symptoms followed by microscopic assay and culture-based morphological aspects [21,22,23]. Currently, pathogen detection is mainly based on nucleic acid hybridization or PCR technology targeting DNA or mRNA. DNA-based assays are more straightforward than mRNA-based assays, and therefore, are more frequently used. In mRNA-based detection, genomic DNA is digested by RNase-Free DNase and reverse transcribed to cDNA for real-time PCR amplification [24]. The higher stability of DNA than mRNA ensures DNA-based methods can yield positive results from inactive pathogens [21,25,26].

PCR techniques, including conventional PCR, real-time PCR, multiplex PCR, etc., provide high sensitivity and specificity for the detection of plant pathogens [27,28,29,30] and have been widely accepted by the plant pathology community as suitable and rapid assays [31,32,33]. In previous years, two real-time PCR assays have advanced the detection and identification of *R. solani* AG-3 [34]. In these assays, primers and probes were designed from a single region, i.e., the rDNA ITS or the β-tubulin gene [18]. To yield consistent results, only small amplicons (50-150 bp) can be used for the real time-PCR assays. 

Sensitivity and accuracy are key factors for molecular detection of fungal pathogens and crucial for implementing molecular detection strategies in plant disease management decisions [35,36,37]. In this context, multiplex PCR technology can provide a better solution than molecular detection based on a single gene. Multiplex PCR amplifies several parts of a pathogen’s genome in the same reaction by mixing multiple primer sets to produce different amplicons specific for different parts of the genome [35,38,39,40]. As a result, this assay is more accurate, especially for highly diverse and rapidly evolving pathogens, and time saving with a lower operational cost. The primers and amplification conditions used in this assay must be carefully designed and properly evaluated by electrophoresis so that all primers work at the same annealing temperature, and amplification products must be of different molecular sizes [41,42,43]. Therefore, the objectives of this research were to establish a sensitive, rapid, and specific multiplex PCR assay for accurate detection of *R. solani* AG-3 and to apply multiplex PCR technology for the detection of *R. solani* AG-3 in soil and potato tubers. 

## 2. Results

### 2.1. Detection of R. solani AG-3 in Single PCR and Multiplex PCR

**Figure 1:**
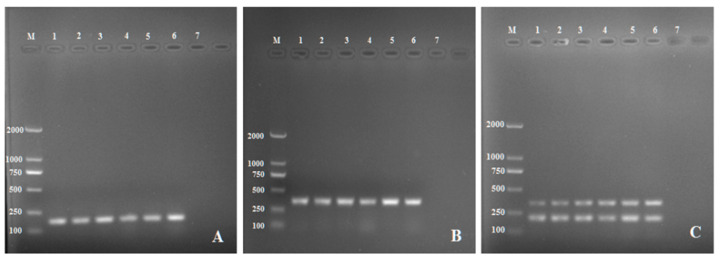
Single PCR with the primer pair RsE-F/R (**A**), (RsE-F/R) for (**B)** and (**C**) Multiplex PCR assay with the two primer pairs, RsP-F/R and RsP-F/R. Lane M: DL 2000 DNA marker; Lane1-6: *Rhizoctonia solani* AG3; Lane 7: Negative control (ddH_2_0).

The genome alignment results showed that endopolygalacturonase (PG1) sequences generated from the six *R. solani* AG-3 isolates were 95.05%, 97.03%, 97.03%, 94.12%, 96.08%, and 95.03% identical to sequence (KF620111.1) downloaded from GenBank with an average identity of 95.72% while PDX1 sequences generated from the six *R. solani* AG-3 isolates were 97.78%, 99.34%, 96.85%, 98.06%, 97.73% and 98.68% identical to the sequence KF934129.1 downloaded from GenBank with an average identity of 97.3%. Genomic DNAs from the six *R. solani* AG-3 isolates were amplified by single PCR and multiplex PCR with two primer pairs (RsE-F/R and RsP-F/R). The results showed that all six isolates were successfully amplified, generating a clear and reproducible 151 bp or 342 bp band by the single PCR for primer pairs RsE (Figure 1A) or RsP (Figure 1B), respectively, and both bands by the multiplex PCR (Figure 1C).

### 2.2. Verification of Primer Specificity by Multiplex PCR

**Figure 2:**
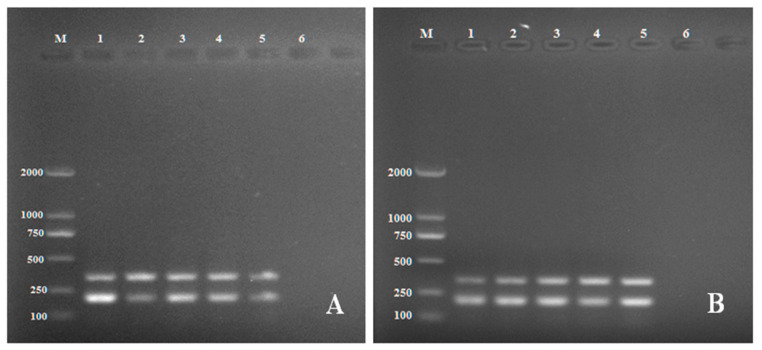
Electrophoresis gels showing the PCR products of *R. solani* with the multiplex primers: (**A**) the annealing temperature optimization. Lane 1: 49 °C, Lane 2: 51 °C, Lane 3: 53 °C, Lane 4: 55 °C, Lane 5:57 °C, Lane 6: Negative control (ddH_2_0) and (**B**) the primer concentration optimization. Lane M: DL 2000 DNA marker Lane 1; 160 nM, Lane 2: 200 nM, Lane 3: 240 nM, Lane 4: 320 nM, Lane 5: 400 nM, Lane 6: Negative control (ddH_2_0) and Lane M: DL 2000 DNA marker.

**Figure 3:**
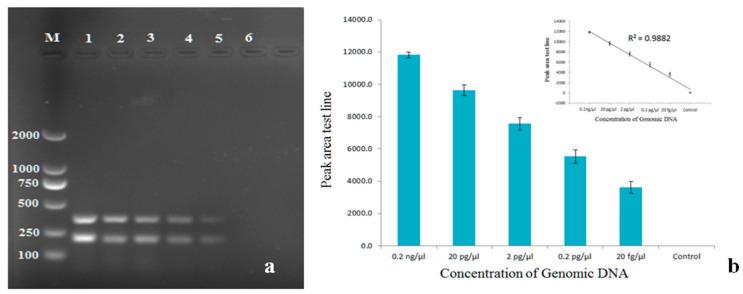
Sensitivity of the multiplex PCR: (**a**) Agarose gel electrophoresis of the amplification products. Lane M: DL 2000 DNA marker Lane 1: 0.2 ng/ μL. Lane 2: 20 pg/ μL. Lane 3: 2 pg/μL. Lane 4: 0.2 pg/μL. Lane 5: 20 fg/μL. Lane 6: negative control. (**b**) Histogram presenting the corresponding peak area of the electrophoretic bands. The inset was a calibration curve for this detection method and the correlation (R^2^) value was 0.9882.

**Figure 4:**
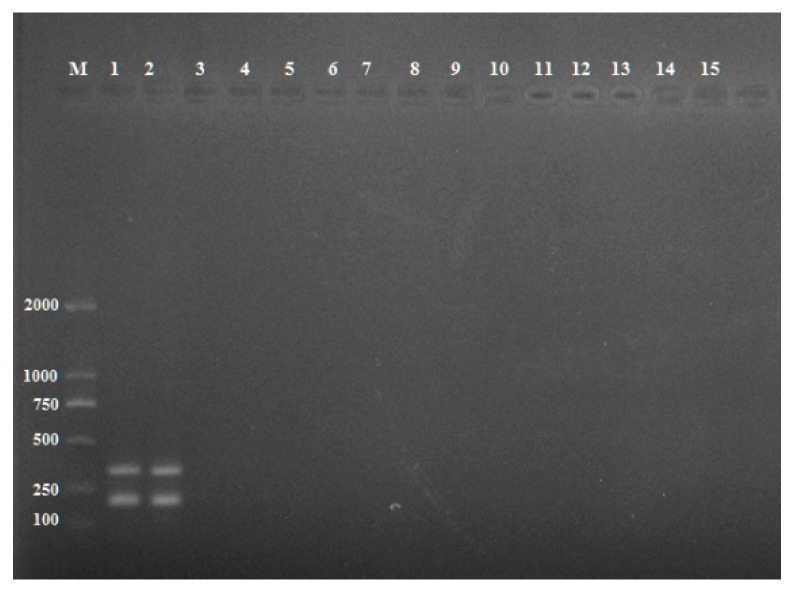
Specificity evaluation of the multiplex PCR. Lane M: DL 2000 DNA marker; Lane 1: *Rhizoctonia solani* AG-3; Lane 2: *Rhizoctonia solani* AG-3; Lane 3: *Rhizoctonia solani* AG-1; Lane 4: *Rhizoctonia solani* AG-4; Lane 5: *Alternaria alternata*; Lane 6: *Alternaria alternata*; Lane 7: *Alternaria alternata*; Lane 8: *Alternaria tenuissima*; Lane 9: *Alternaria tenuissima*; Lane 10: *Alternaria tenuissima*; Lane 11: *Phytophthora nicotianae*; Lane 12: *Phytophthora capsici*; Lane 13: *Phytophthora sojae*; Lane 14: *Alternaria solani*; Lane 15: negative control (ddH_2_0).

Diagnostic specificity is defined as a measure of detection effectiveness thus the specificity of the primers in multiplex PCR was tested using species closely related to *R. solani* AG3, such as *R. solani* AG-1 and AG-4 and other fungal pathogens (Table 1). Primer specificity for target genes *PG1* and *PDX1* was tested under the optimum conditions. The intensities of the resulting size-specific bands were analyzed by peak areas of gel electrophoresis. The optimum annealing temperature was 53 °C (Figure 2A) as indicated by the highest mean peak area of the two amplicons and the optimum primer concentration was 240 nM (Figure 2B). Sensitivity testing showed *R. solani* AG-3 was able to be detected at a concentration of 20 fg/μL DNA (Figure 3). The primers did not produce reliable amplicons when DNA concentrations of *R. solani* AG-3 were further reduced and did not amplify other *R. solani* AG groups and fungal pathogens, indicating the primers are specific for *R. solani* AG-3 and 20 fg/μL is likely the minimum DNA concentration required to detect the pathogen (Figure 4).

### 2.3. Detection of Rhizoctonia solani in Artificially Infected Soils and Tubers by Multiplex PCR

**Figure 5:**
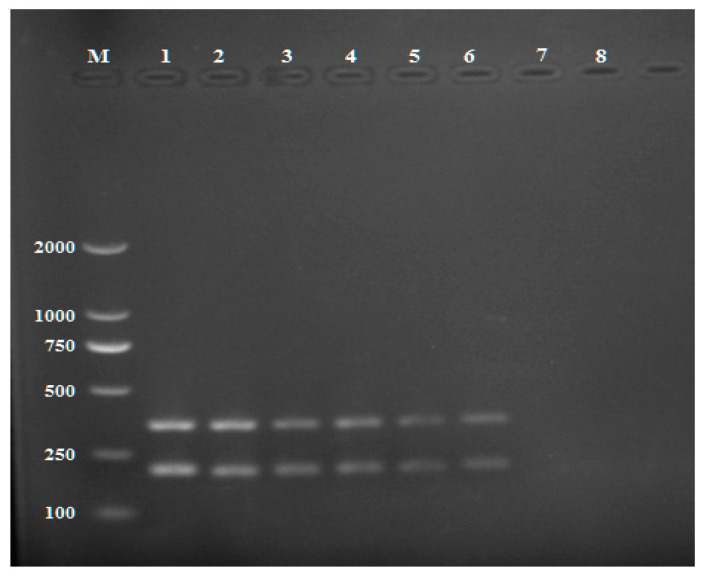
Detection of *Rhizoctonia solani* AG-3 from artificially infected soil. Lane M: DL 2000 DNA marker; Lane 1 and 2: 6 × 10^−3^ g sclerotia; Lane 3 and 4: 6 × 10^−4^ g sclerotia; Lane 5 and 6: 6 × 10^−5^ g sclerotia; Lane 7: sterile un-inoculated soil, Lane 8: negative control (ddH_2_0).

*Rhizoctonia solani* develops living structures, such as spores or melanized hyphae that can exist in the soil for several years even without a host. Rapid and accurate detection of *R. solani* in the soil can increase the management effectiveness and minimize field infection. *R. solani* in soil artificially infected with the pathogen was successfully detected using the multiplex PCR (Figure 5). Concentrations as low as 6 × 10^−5^ g *R. solani* sclerotia in soil were able to be detected by the PCR approach but the further reduction of sclerotia in soil did not produce reliable amplicons.

**Figure 6:**
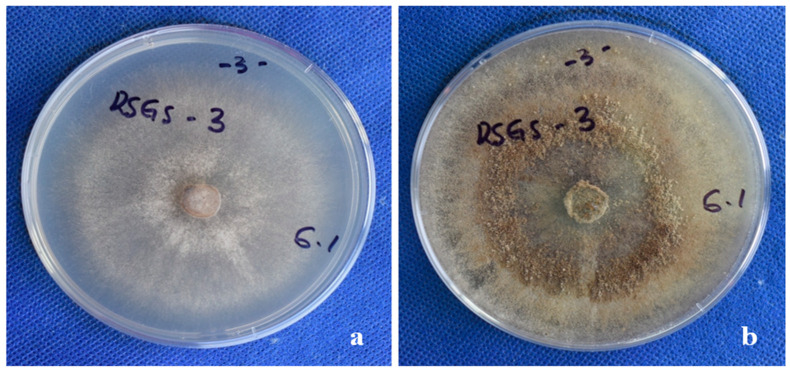
The morphology of *R. solani* colonies observed in the early (7th day after inoculation, (**a**)) and late (**b**) stage of development.

**Figure 7:**
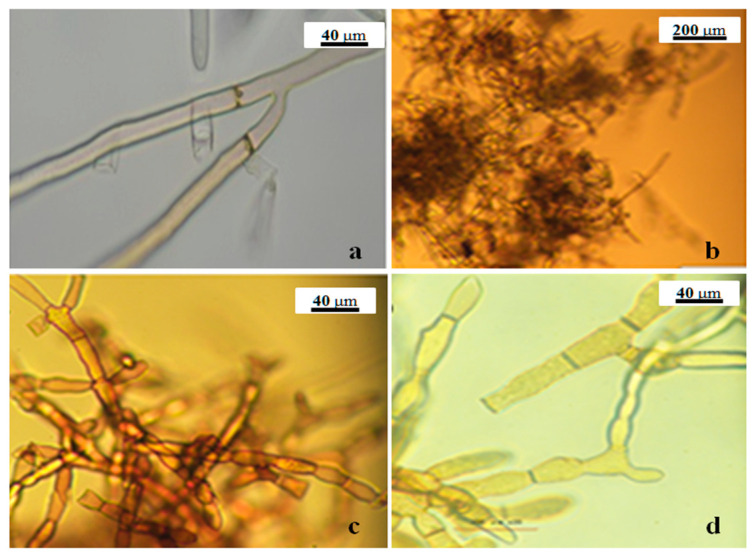
The morphology of *Rhizoctonia solani* recovered from potato tubers; (**a**) dolipore septum, (**b**) hyphal lysis, (**c**) sclerotium and (**d**) modiolid cell.

Before the PCR test, the identity of the pathogen was confirmed morphologically by visual inspection and microscopic observation. The pathogen recovered from infected soil produced typical characters of *R. solani,* including buff mycelium in an early stage and brown mycelia in the late stage (Figure 6), dolipore septum, and hyphal lysis, sclerotium, and modiolid cell figure (Figure 7)

**Figure 8:**
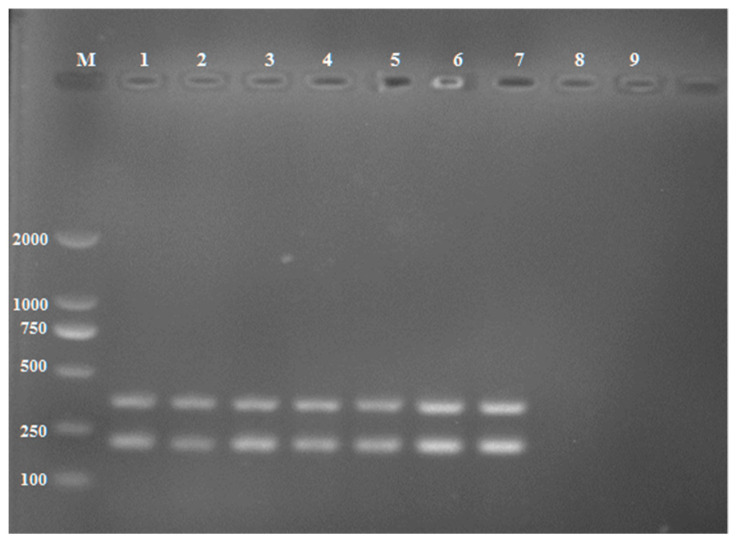
Detection of *Rhizoctonia solani* AG-3 from potato tubers by the multiplex amplification of the pathogen developed. Lane M: DL 2000 DNA marker; Lane 1–7: Infected tubers, Lane 8; Healthy tuber, Lane 9: Negative control (ddH_2_0).

*R. solani* was also successfully detected in potato tubers artificially inoculated with one sclerotium. As for soil detection, morphological confirmation was also performed for the pathogen recovered from potato tubers. The expected bands were amplified from DNA extracted from the infected tubers (Figure 8). No amplicons were produced from DNA extracted from healthy tubers. The results showed that the multiplex PCR assay can detect the pathogen from a small piece of potato tissue. Therefore, we believe that the PCR technique can play a significant role in managing the potato disease caused by AG-3.

## 3. Discussion

Pathogenic fungi are the causal agent of most dangerous plant diseases, leading to severe biological and economic losses of many important crops worldwide. A highly relevant strategy to control plant diseases is the early detection of pathogens in planting materials [35,44,45]. *R. solani* is a soil-borne fungal pathogen causing disease in potatoes. The disease can have destructive effects on potato yield and quality. Accurate and rapid detection of the pathogen is a milestone in studying the epidemiology of the disease and an essential step leading to its successful management. Previously, the disease was mainly diagnosed by morphological detection of *R. solani* based on cultural characteristics of mycelium and microscopic observation. In this study, we established a multiplex PCR by standardizing critical parameters for molecular detection, complementary to the quantitative real-time PCR approach of detecting AG-3 from soil and potato tissues using internal transcribed spacers (ITS1 and ITS2) developed previously [34]. 

Analysis of sequences through BLAST showed very high sequence identity with references downloaded from GenBank, confirming that the six isolates of *R. solani* belong to AG-3. Developing efficient multiplex PCR detection of a pathogen needs technical planning and experimentation to optimize reaction conditions [46,47]. These optimizations aim to reduce nonspecific interactions, whereas primer pairs for different genomic targets must share the same amplification conditions, such as annealing temperature [42]. Our evaluation revealed that the optimal annealing temperature for the multiplex PCR assay was 53 °C (Figure 2). 

Sensitivity, reproductivity, and specificity are three main factors that determine the effectiveness of multiplex PCR detection of plant pathogens. The successful amplification of the pathogen from a small amount of DNA supports the high sensitivity of our approach. The result showed that it was able to successfully detect the pathogen at 20 fg/μL of DNA concentration (Figure 3). Furthermore, the use of commercial soil-extraction kits could increase the sensitivity of detection. Previous assays require a minimum concentration of 5 × 10^−3^ g sclerotia/g soil for detection [34]. The pathogen materials required for successful detection from potato tissues in our methods are significantly lower than the previous assays. 

Reproducibility of the sensitivity was determined by the three tests shown on the histogram, and the lowest level of DNA required for detection was 20 fg/μL (Figure 3b). The positive correlation between the peak area of detection and the concentration of genomic DNA was statistically significant (R^2^ = 0.9882). A definitive sensitivity test was not performed in the previous publication [34], but is critical for diagnosis, as it is necessary for determining the lowest quantity of DNA that allows us to detect infection during the early stages.

Specificity is a practical key to the detection of infectious plant pathogens. Our primers only amplify DNAs from AG-3 but not from other fungal pathogens tested (Figure 4, Table 1). Like other PCR detection of the pathogen, we were unable to collect all 13 AGs and include them in this study, but our protocol can unequivocally distinguish AG3 from AG1 and AG4. The use of two specific primer pairs has proved to be a successful strategy for improving a diagnostic test to detect many pathogens [35] and our protocol will add additional resources for effective detection and control of this plant pathogen in agriculture. In addition, the most crucial step in establishing a multiplex PCR assay is the primer design which usually defines the specificity and sensitivity of the assay. The specificity and sensitivity of multiplex PCR technology for detecting *R. solani* AG-3 were high, and the assay could be completed within 1.5 hours. Therefore, this establishment of multiplex PCR provides a rapid and reliable method and will be helpful for detecting *R. solani* AG-3.

In conclusion, the multiplex PCR assay we established is a suitable, sensitive, and efficient approach to detecting *R. solani.* It is also less expensive and less time-consuming than other molecular detection currently used with good specificity, accuracy, and sensitivity. For example, it is less restrictive in terms of reagent and product size requirements compared to real-time PCR and has higher accuracy than single-gene PCR because the multiplex PCR analysis can simultaneously amplify different parts of the pathogen’s genome, thereby avoiding potential glitches arising from mutations in target genes, such as ITS regions of nuclear ribosomal DNA [48]. This kit will benefit the early detection of *R. solani* in potatoes and timely prevention of the disease.

## 4. Materials and Methods

### 4.1. Source of Isolates and Morphological Identification

Fungal and oomycete species used in this study are listed in Table 1. *Rhizoctonia solani* samples were collected from different geographic areas in China and were stored on potato dextrose agar (PDA) or V8 agar slants. Other fungal and oomycete pathogens were available in the laboratory. Tubers samples were washed under tap water to remove debris of the soil. The affected tissue was placed on water agar and incubated at 25 °C for seven days. Morphological characteristics of infected tubers were assessed by visual observation. Afterward, the pathogen identification was microscopically confirmed as previously described [4].

### 4.2. DNA Extraction

Isolates were grown on a cellophane disc placed on PDA media for seven days, and mycelia were harvested; 300 mg of mycelia were then transported into sterile 2 ML centrifuges tubes and lyophilized by a vacuum freeze dryer (Alpha1-2, Christ, Germany). Dried mycelia were ground into powder by a mixer mill (MM4400, Resch, Germany). Total genomic DNA was extracted under the instruction of a DNA extraction Kit (D3390-02, Omega Bio-Tek, and Beijing, China). To ensure DNA samples were of sufficient quality, concentrations were determined on a NanoDrop ND-1000 spectrophotometer (Nano Drop Technologies Inc., Wilmington, DE).

### 4.3. Primer Design

PG1 and PDX1 (Endo-Polygalacturonase1 and Pancreatic and duodenal homeobox1) are highly conserved genes [49]. The nucleotide sequences of PG1 and PDX1 genes in *R. solani* AG-3 are expected to have a high degree of similarity to other fungal species and be closely related to other *R. solani* anastomosis groups. Degenerate primers were designed from pg1 (KC291405.1) and *PDX1* (KF620111.1) sequences of *R. solani* AG-3 downloaded from NCBI and used in PCR reactions to amplify putative PG1 and *PDX1* genes in the genomic DNA of *R. solani.* Sequence regions conserved for isolates of AG-3 but distinct from other AGs were identified using Primer 6.0 software. Two pairs of specific primers (Table 2) were designed with potential specificity to AG-3.

### 4.4. Detection of Rhizoctonia Solani by Single PCR 

*R. solani* was assessed in single PCR for the sets of pair primers (Table 2). Reaction was performed in a total volume of 25 µL in a micro-tube containing 2 μL of deoxynucleotide triphosphate (dNTPs), 0.1 μL of Taq DNA polymerase (5 U/μL), 2.5 μL of 10 × PCR buffer, 1 μL of forwarding primer (10 μM), 1 μL of reverse primer (10 μM), 10^−6^ ng of DNA template and 17.4 μL of ddH_2_O (deionized water). For the negative control, distilled water was used as a template. The amplification was carried out in an Eppendorf AG thermal cycler with the following condition: denaturation at 95 °C for 2 min, 35 cycles each consisting of the next step: denaturation at 95 °C for the 30 s, annealing at 52 °C for 45 s, extension at 72 °C for the 60 s and final extension 72 °C for 5 min, The PCR products with expected amplicons were visualised by in 1 × TAE electrophoresis agarose gel at 120 V in 45 min. The PCR products were sequenced by a company to obtain the sequences. BLAST against the sequences of known anastomosis groups (AGs) in NCBI was performed to confirm the species identity of the materials we used.

### 4.5. Multiplex PCR Detection of R. solani in Artificially Infected Soil

Soil samples (loam soil) collected from a field that was not planted with potatoes for the past five years were air-dried for three days and sterilized by autoclaving at 121 °C for 15 minutes prior to artificial inoculation. To obtain sclerotia for inoculation, *R. solani* AG-3 was grown on PDA with sterile cellophane slips for two weeks. Sclerotia harvested were lyophilized and ground with a mixer mill (MM400, Retsch, Germany). Artificial inoculation was done using 0.25 g of soil mixed with powder of the sclerotia calibrated to 6 × 10^−3^ g sclerotia/g soil, 6 × 10^−6^ g sclerotia/g soil, and 6 × 10^−5^ g sclerotia/g soil. The DNeasy^R^ power soil^R^ kit was used to extract DNA from the artificially infected soil according to the manufacturer’s instructions. Multiplex PCR was set up in a total volume of 25 µL containing 2 µL of deoxynucleotide triphosphate (dNTPs), 0.1 µL for Taq DNA polymerase (250 U), 2.5 µL of 10× PCR buffer, 0.6 µL forward primer (10 µM), 0.6 µL of reverse primer (10 µM), 1 µL of DNA template, 1 µL of un-inoculated soil and 17.4 µL of ddH_2_O (deionized water). For negative control, the template was replaced by distilled water. As described above, multiplex PCR was carried out in an Eppendorf AG thermal cycle.

### 4.6. Multiplex PCR Detection of R. solani in Infected Tubers 

Potato tubers with symptoms of black scurf were rinsed under tap water to remove the soil, and then the tubers were thinly peeled using a sterilized scalpel to take a small piece of tissue (1 sclerotium cm^2^). The tissue was then transferred into 2 mL sterilized centrifuge tubes and lyophilized with a vacuum freeze dryer (Alpha1-2, Christ, and Germany) for 6 h. The infected tissue was ground to powder with a mixer mill (MM400, Retsch, Germany). Total genomic DNA of the infected tubers was extracted using Easy Pure Plant Genomic DNA according to the manufacturer’s instructions. Multiplex PCR was set up in 25 µL total volume containing 2 µL of deoxynucleotide triphosphates (dNTPs), 0.1 µL of Taq DNA polymerase, 2.5 µL of 10 × PCR buffer, 1 µL forward primer (10 µM), 1 µL of reverse primer (10 µM), 1 µL of DNA template extracted from the infected tubers, or healthy tubers (the control) and 17.4 µL of ddH_2_O (deionized water). As described above, the multiplex PCR was performed in an Eppendorf AG thermal cycle.

### 4.7. Optimization of Multiplex PCR

Multiplex PCR was conducted by mixing more than one pair of primers. To obtain an efficient reaction and ensure good amplification, reaction conditions, such as annealing temperature and primer condensation must be optimized for the different primer pairs. In this study, we evaluated these two parameters under different reactions. The selected annealing temperature ranged from 49 °C to 57 °C. The previous results [49] showed that lowering annealing temperature by 4–6 °C was required for different loci to be co-amplified in multiplex mixtures. Because one pair of our primers (RsE151-R and RsE151-F) has a Tm (melting temperature) of 53 and 52.6 °C and another pair (RsP342-R and RsP342-F) has 55.2 and 54.9 °C, respectively, reducing 4–6 °C from the melting temperature will allow them to bind. In our study, we chose equimolar primer concentrations ranging from 160 nM to 400 nM. Further studies showed that the primer concentration ranged from 40 nM to 500 nM resulting in either high copy number or low complexity of DNA [50].

### 4.8. Sensitivity Assay

Sensitivity is a critical factor for pathogen detection. We evaluated the sensitivity of multiplex PCR with a series of DNA concentrations extracted from the target pathogen *R. solani* AG-3. DNA concentration was measured by NanoDrop ND-100 spectrophotometer (Nano Drop Technologies Inc, Wilmington, DE) followed by serial 10-fold dilutions of genomic DNA concentration (ranging from 0.2 ng/μL to 20 fg/μL) for the determination of the lowest concentration required for multiplex PCR.

## Figures and Tables

**Table 1:** Fungal and oomycete isolates used in this study.

Code	Host	Origin	Anastomosis Group/Species
AG-3	Potato	Gansu Province	*Rhizoctonia solani* AG-3
AG-1	Rice	Yunnan Province	*Rhizoctonia solani* AG-1
AG-4	Cotton	Hubei Province	*Rhizoctonia solani* AG-4
PSI	Glycine max	Fujian Province	*Phytophthora sojae*
PCI	*Capsicum annuum*	Fujian Province	*Phytophthora capsici*
PNI	*Nicotiana tabacum*	Chongqing Province	*Phytophthora nicotianae*
AA	Potato	Fujian Province	*Alternaria alternata*
AT	Potato	Fujian Province	*Alternaria tenuissima*
AS	Potato	Shandong Province	*Alternaria solani*

**Table 2:** DNA sequences of the specific primers used to amply *Rhizoctonia solani* and their amplicon sizes.

Target Gene	Primer Name	Sequence (5’-3’)	Amplicon Size (bp)
Endopolygalacturonase	RsE-F	GCACTATCTCGTCGTTGA	151
	RsE-R	ACTCCGAACTTGACATCTC	
Pyridoxine biosynthesis protein PDX1	RsP-F	GCGTTTGCCAGTTGTCAG	342
	RsP-R	CACATAGTCAGCCCAACCA	

## Data Availability

Not applicable.
